# Dietary selenium intake among Ethiopian children in areas known for selenium spatial variability

**DOI:** 10.3389/fnut.2023.1250002

**Published:** 2023-10-16

**Authors:** Kaleab Hailu, Edward J. M. Joy, Elaine L. Ferguson, Elizabeth H. Bailey, Lolita Wilson, Kenneth Davis, Martin R. Broadley, Dawd Gashu

**Affiliations:** ^1^Center for Food Science and Nutrition, Addis Ababa University, Addis Ababa, Ethiopia; ^2^Department of Food Science and Applied Nutrition, Addis Ababa Science and Technology University, Addis Ababa, Ethiopia; ^3^Department of Population Health, London School of Hygiene and Tropical Medicine, London, United Kingdom; ^4^Sustainable Soils and Crops Department, Rothamsted Research, Harpenden, United Kingdom; ^5^School of Biosciences, University of Nottingham, Loughborough, United Kingdom

**Keywords:** selenium, mineral spatial variability, food mineral concentration, breast milk selenium, dietary mineral intake

## Abstract

**Introduction:**

There is spatial variability of selenium (Se) in soil and crops in Ethiopia. We assessed the Se content of food items, breast milk, and urine among infants in Ethiopia from two areas with contrasting Se concentrations in soils.

**Methods:**

Dietary Se intakes among children (6–23 months) were evaluated using a weighed food record on two non-consecutive days. Also, spot urine samples from children and breast milk samples from their mothers were collected to determine Se concentration. Selenium concentrations in the samples were analyzed using an inductively coupled plasma mass spectrometer (ICP-MS).

**Results:**

Injera (prepared from teff and mixtures of other cereals) with a legume-based stew were the most frequently consumed foods by the children in both areas, followed by pasta. Overall, the Se concentration (mean ± SD) of food items, breast milk (12.2 ± 3.9 μg/L vs. 3.39 ± 1.5 μg/L), and urine samples (22.5 ± 11.5 μg/L vs. 3.0 ± 1.9 μg/L) from East Amhara were significantly higher than the corresponding samples from West Amhara (*p* < 0.001). The total Se intakes by the study children from East Amhara and West Amhara were 30.2 [IQ _25%_, 14.2; IQ _75%_, 54.1] and 7.4 [IQR _25%_, 4.2; IQ _75%_, 10.6] μg day^–1^, respectively; 31.5% of children from East Amhara and 92% of children from West Amhara were at risk of inadequate Se intakes. Urinary Se excretion accounted for 53 and 39% of daily dietary Se intake in East Amhara and West Amhara, respectively. Dietary Se intake was positively correlated with urinary Se excretion in East Amhara (*r* = 0.56; *p* < 0.001) but not among samples from West Amhara (*r* = 0.16; *p* ≥ 0.05), suggesting greater physiological Se conservation in a state of deficiency.

**Conclusion:**

There is spatial variability of Se in foods, breast milk, and urine in Ethiopia, suggesting the need for implementation of targeted agronomic interventions that enhance Se concentrations in the edible portion of plant foods.

## Background

Micronutrient deficiency (MND), defined as the inadequate intake of vitamins and minerals that are needed in small amounts by the body, is an important global health issue affecting 2 billion people worldwide (1). The most common MNDs include iron (Fe), iodine (I), folate, vitamin A, and zinc (Zn). Deficiencies of these micronutrients are linked to impaired physical and mental growth and compromised immunity, productivity, and survival (2). Selenium is also of interest due to its importance in many physiological processes and biological functions such as antioxidant defense systems, DNA synthesis, fertility, and reproduction and immune function (3). Selenium deficiency has been associated with an increased incidence of chronic non-communicable diseases, such as cancer, cardiovascular diseases, type 2 diabetes, and immune deficiencies (4), and implicated to negatively affect normal thyroid metabolism (5, 6) and impaired cognitive performance (7). A higher mortality risk from COVID-19 has also been reported among Se-deficient individuals (8).

Soil physico-chemical characteristics (e.g., pH and organic matter content) and its Se content control grain Se concentration, which determines Se exposures among humans and grazing livestock (9, 10). Populations dependent on subsistence agriculture are particularly vulnerable to Se deficiency when local soils are low in plant-available Se. Previously, we reported the spatial variability of Se in agricultural soils, cereal grains (11, 12), and human serum (13) in Ethiopia. There is also strong evidence that the Se status of livestock differs in two areas in the Amhara region that are a short distance apart (East Amhara and West Amhara, Ethiopia) (14). A similar pattern was observed among children in Amhara with a high prevalence of biochemical Se deficiency in West Amhara and little or no deficiency in East Amhara (7). This contrast could arise due to differences in food choices and feeding practices as affected by agroecology (15). In the present study, we evaluated the feeding practices and dietary Se intakes of young children (aged 6–23 months) from Amhara regions known for their contrasting soil, crops, livestock, and human serum Se concentrations.

## Materials and methods

### Study area and study population

The study was conducted in the South Wollo (East Amhara) and West Gojjam (West Amhara) zones of the Amhara region where contrasting Se concentrations in soils, food crops, and human serum samples have been observed (12, 13). Three woredas (Dessie Zuriya, Kalu, and Tehuledere) from the South Wollo zone and two woredas (Bure Zuriya and Jabi Tenna) from the West Gojjam zone were selected based on proximity to facilities and road access for proper sample collection, handling, and storage. In the selected districts, eight villages from South Wollo and seven villages from West Gojjam were randomly selected. Lists of households were obtained from health posts. With the help of health extension workers and available records, households with at least one child between the age of 6 and 23 months who had resided in the area for at least 6 months before the study’s recruitment period were listed, and 75 households were randomly selected from each zone. In cases where more than one eligible child were available in a household, a lottery method was used to pick a child. An additional three households (not originally selected) from selected villages in West Amhara were included because a household member made a request to participate in the study.

### Study design

A cross-sectional survey was conducted from March to May, 2020. In this survey, dietary, anthropometric, biochemical, and questionnaire data were collected from the children or their parents, and food samples were collected. Data collectors with a first degree in health, agriculture, or social sciences who were familiar with the local area were recruited and trained in the standard procedures for administering the questionnaire and dietary intake assessment. They were initially trained in the classroom, including on standardized methods for questionnaires, weighed food record data collection, and the recording procedure, followed by pilot testing in the field. Nurses from local health facilities took the anthropometric measurements after refresher training and they were required to pass an evaluation of their accuracy and precision in the anthropometric measurements.

### Socio-demographic and anthropometric characteristics

Information on the socio-demographic characteristics, occupation, and education level of mothers/guardians were collected using a structured and pre-tested questionnaire. Measurements of both child weight and length were taken in triplicate. The weight of each child was measured with light clothes on, in the arms of an adult participant to the precision of 0.1 kg on an electronic battery-powered digital balance (UNICEF SECA 874 U, UNICEF Supply Division, Copenhagen, Denmark). The weight of adults was measured using the same balance and subtracted from the total weight (adult + child). The balance was calibrated at least twice a day. Child recumbent length was measured to the nearest 0.1 cm using a UNICEF measuring board (UNICEF Supply Division, Copenhagen, Denmark). The Z-scores for length-for-age (LAZ), weight-for-age (WAZ), and weight-for-length (WLZ) were calculated using WHO multicenter growth reference data (16).

### Dietary intake assessment and food sample collection

A weighed food record was collected on two non-consecutive days to estimate the food and beverages consumed by each participating child from the time the child woke up in the morning until they went to bed for the night (17). Each data collector was assigned to visit two nearby households in a day. All days of the week were proportionately represented to account for any day-of-the-week effect on food intake (17). All foods and beverages consumed by a child were weighed using a digital balance (SF-400A). Accuracy of the balance was checked using a known weight (500 g) prior to the weighing the child’s food intakes. Serving plates/cups were dried with a dry cloth and their weight was recorded prior to serving food to the child (W1). Mothers/guardians were asked to place foods on the weighed plates/cups in amounts exceeding the usual serving size and the serving plates/cups were weighed with foods (W2). Any leftover food was weighed (W3) and collected in closed polyethylene zipper bags and wrapped with aluminum foil prior to storage in a refrigerator. The weight of food eaten by the study child was calculated by difference as W = (W3-W1)–(W2-W1). The weight of any spillage was estimated using spoons and was deducted from W2. Data collectors stayed outside when child was fed to minimize distractions.

### Breast milk collection

Breast milk was collected when mothers felt comfortable using a manual breast milk pump. Before milk collection, the nipples and areolas of the breasts were cleaned with deionized water (18). Samples were transported and temporarily stored at −20°C in the nearest health facility, (approximately 1–3 h after collection) before being transported to the Center for Food Science and Nutrition Laboratory, Addis Ababa University, in cold storage.

### Urine collection

Spot urine samples of children were collected on the first day using adhesive pediatric urine bags after cleaning the genital regions. The urine samples were transferred to urine tubes and tightly sealed, stored in ice boxes, and transferred to a freezer in nearby health facilities for storage at −20°C. The samples were then transported to Addis Ababa University in cold storage and stored at −80°C. Aliquots of frozen urine samples were shipped on dry ice to the University of Nottingham, UK, under the material transfer agreement (MTA) for elemental analysis.

### Sample preparation

The food samples (with the exception of cow milk and breast milk) were freeze dried using a Lyophylizer (Mini Lyodel, India) at the Center for Food Science and Nutrition Laboratory, Addis Ababa University. Dried samples were ground in an acid-cleaned mortar and pestle and 20 g of each sample was packed in to airtight bags. Aliquots of 15–20 mL of cow milk and breast milk samples were transferred to labeled containers and frozen. Samples were transported on dry ice to the University of Nottingham, under the MTA for multi-element analysis.

Dried and ground food samples (ca. 0.4 g) and breast milk and cow milk samples (1 mL) were digested in concentrated nitric acid (68% HNO_3_ Primar Plus™ for Trace Metal Analysis, Fisher Scientific) using a hotplate digestion system (Anton Parr-PFA coated graphite hot block). The digests were diluted with Milli-Q^®^ (MQ) water (18.2 MΩ cm) in 1 to 10 ratios prior to analysis. Operational blanks (*n* = 14) were digested alongside samples in each batch. Samples of certified reference material, National Institute of Standards and Technology (NIST) wheat flour SRM 1567b and ERM-BD150 dried milk powder, were included in digestion batches of food and milk samples, respectively.

Urine samples were diluted in 2% Trace Element Grade HNO_3_ prior to analysis. Seronorm Trace Elements Urine L-1 and L-2 (SERO, Billingstad, Norway) were prepared and analyzed alongside the urine samples.

### Se analysis in food, urine, and breast milk samples

Multielement analysis was conducted using ICP-MS (Thermo-Fisher Scientific iCAP-Q, Thermo Fisher Scientific, Bremen, Germany). Samples were introduced (flow rate 1.2 mL min^–1^) from an autosampler (Cetac ASX-520) incorporating an ASXpress™ rapid uptake module through a perfluoroalkoxy (PFA) Microflow PFA-ST nebulizer (Thermo Fisher Scientific, Bremen, Germany). Sample processing was undertaken using Qtegra™ software (Thermo-Fisher Scientific) utilizing external cross-calibration between pulse-counting and analogue detector modes when required. Selenium (m/z 78) was measured in hydrogen (H_2_) reaction mode to reduce polyatomic interferences. Calibration standards (Claritas-PPT grade CLMS-2, SPEX Certiprep Inc., Metuchen, NJ, USA) for Se were prepared in the range 0–100 μg L^–1^ (0, 20, 40, and 100 μg L^–1^). Internal standards, used to correct for instrumental drift, were introduced to the sample stream on a separate line (equal flow rate) via the ASXpress unit. Internal standards typically included combinations of Sc (10 μg L^–1^), Ge (10 μg L^–1^), Rh (5 μg L^–1^), and Ir (5 μg L^–1^). The matrices used for internal standards, calibration standards, and sample diluents were 2% v/v trace analysis grade HNO_3_ (Fisher Scientific, UK) with 4% methanol to enhance the ionization of Se.

The limit of detection (LOD, 3 × SD blank digest) for Se in food samples was 0.0039 mg/kg (*n* = 14), in milk samples was 0.0068 mg/kg (*n* = 10), and in urine was 0.013 μg/L (*n* = 10). The limits of quantification (LOD, 10 × SD blank digest) were 0.013 mg/kg, 0.023 mg/kg, and 0.043 μg/L for food, milk, and urine samples, respectively. Recovery of Se was 87% (*n* = 4) for NIST wheat flour SRM 1567b and 91% (*n* = 3) for ERM-BD150 Milk Standard. Seronorm Trace Elements Urine L-1 (*n* = 2) and L-2 (*n* = 2) gave average values of 19.0 μg/L (acceptable range 8.3–19.5 μg/L) and 78.7 μg/L (acceptable range 41.9–98.3 μg/L).

### Specific gravity analysis for urine samples

Specific gravity was measured using a temperature-corrected refractometer (PAL-10S, Atago, Japan) on 300 μL samples to enable correction of Se concentrations for hydration status as described in Phiri et al. (19).

### Statistical analysis

The WHO Anthro software was used for analysis of anthropometric data. Length-for-age, weight-for-age, and weight-for-length were used to classify the study children into categories of nutritional status using the WHO Multicenter Growth Reference (16). Children below -2 height-for-age z-score (HAZ), -2 weight-for-age z-score (WAZ), and -2 weight-for-height z-score (WHZ) were classified as stunted, underweight, and wasted, respectively. The Se intake from breast milk was calculated using the assumption of average daily breast milk intake of 641 mL/day for infants between 6 and 8 months, 598 mL/day for infants between 9 and 11 months, and 533 mL/day for infants between 12 and 23 months (20). The amount of food and the Se content of the foods and breast milk consumed by the study participants in the 24 h preceding data collection were used to calculate daily Se intake. Age-aggregated reference values (15 μg/day, 4–47 months old children) for adequate Se intake by Kipp et al. (21) were used to determine adequacy. Statistical analysis was performed using SPSS for Windows (v18). Normality of data was checked using the Kolmogorov–Smirnov test before conducting the correlation and comparison analysis. Pearson correlation and Student’s t-test were conducted on the log-transformed data to conform normality to study bivariate correlations and the comparison of variables, respectively.

### Ethical review

This study was conducted according to the guidelines laid down in the Helsinki Declaration for all procedures involving human subjects. The study was approved by the National Research Ethics Review Committee at the Ministry of Science and Technology, Ethiopia (Reference 3.10/433/06), and Addis Ababa University Institutional Review Board (Reference CNSDO/450/10/2018). Written informed consent was obtained from all mothers or guardians.

## Results

### Socio-demographic and anthropometric characteristics

The socio-demographic characteristics of the study households are presented in Table 1. The mean age of mothers/caretakers was 27.6 years in East Amhara and 29.4 in West Amhara. The majority of the mothers/caretakers had primary education. The percentages of mothers who were breast feeding at the time of data collection were 76% in East Amhara and 95% in West Amhara. Some 81.3% of mothers in East Amhara and 62% in West Amhara reported starting complimentary feeding of their child at 6 months. The study children had a male to female ratio of 1.04: 0.95. Children from East Amhara had comparable mean age with those from West Amhara (15.3 months vs. 14.5 months). Stunting was prevalent both in East Amhara and West Amhara. In addition, both underweight and wasting were prevalent among children from West Amhara (Table 2).

**TABLE 1:** Socio-demographic characteristics of study households and infants.

Variable		East Amhara, *n* (%)	West Amhara, *n* (%)
**Mothers’ educational status**			
	Attended informal education and can read and write	27 (37.1)	47 (60.3)
	≤ 8th grade	29 (39.7)	22 (28.2)
	9th–12th grade	15 (20.5)	6 (7.7)
	≥ 12th grade	2 (2.7)	3 (3.8)
**Marital status**			
	Married	67 (90.5)	71 (91.0)
	Unmarried	2 (2.7)	1 (1.3)
	Divorced	5 (6.8)	5 (6.4)
	Widowed	0 (0.0)	1 (1.3)
**Occupation**			
	Farmer	22 (30.1)	48 (61.5)
	Daily laborer	17 (23.3)	14 (17.9)
	Housewife	24 (32.9)	8 (10.3)
	Other	10 (13.7)	8 (10.3)
**Source of drinking water for the household**			
	Tap water	67 (90.5)	66 (86.8)
	Protected borehole	7 (9.5)	10 (13.2)
**Complementary food preparation training**			
	Yes	0 (0.0)	74 (97.4)
**Complementary food feeding frequency**			
	≤ 5 times	54 (73.0)	66 (85.7)
	> 5 times	20 (27)	11 (14.3)

**TABLE 2:** Anthropometric characteristics of study subjects.

Measured characteristics	East Amhara				West Amhara			
	Minimum	Maximum	Mean (SD)	<-2SD, *n* (%)	Minimum	Maximum	Mean (SD)	<−2SD, *n* (%)
WAZ	−2.8	4.2	−0.3 (1.4)	8 (11.1)	−4.2	2.2	−1.3 (1.17)	15 (19.5)
HAZ	−5.1	2.8	−1.6 (1.8)	28 (38.9)	−4.3	1.9	−1.1 (1.26)	15 (19.5)
WHZ	−3.0	5.2	0.6 (1.5)	1 (1.4%)	−5.2	1.9	−1.1 (1.23)	19 (24.7)

WAZ, weight for age Z-score; HAZ, height for age Z-score; WHZ, weight for height Z-score.

### Dietary and breast milk Se concentration

Injera (prepared from teff and mixtures of other cereals) with legume stew was the most frequently consumed food item by the infants/young children from both areas, followed by pasta (Table 3). The too early (4.1%) or late (21.8%) introduction of complementary feeding was common in both areas. About two-thirds of children from East Amhara and the majority from West Amhara were breastfeeding at the time of data collection.

**TABLE 3:** Most frequent types of complementary food consumed by infants in Northern Ethiopia, 2020.

Food items	East Amhara, *n* (%)		West Amhara, *n* (%)	
	Day 1	Day 2	Day 1	Day 2
Injera with legume-based stew	49 (67.1)	51 (69.9)	54 (69.2)	59 (75.6)
Pasta^‡^	41 (56.2)	37 (50.7)	27 (34.6)	14 (17.9)
Bread	17 (23.3)	19 (26.0)	21 (26.9)	8 (10.3)
Cow’s milk	16 (21.9)	16 (21.9)	5 (6.4)	4 (5.1)
Cereal-based porridge	13 (17.8)	11 (15.0)	40 (51.3)	27 (34.6)

^‡^pasta, macaroni, instant noodles.

Breast milk Se concentration was 12.2 ± 3.9 μg/L in East Amhara and 3.4 ± 1.5 μg/L in West Amhara with a significant difference at *p* < 0.05. The mean Se concentrations of the predominant food types are listed in Table 4. There were significant differences in Se concentrations in injera, gruel/porridge, bread, and breast milk samples between the two areas, while no difference was seen in pasta/macaroni, a food item that is centrally processed and purchased at markets.

**TABLE 4:** Comparison of mean Se concentration (μg/100 g) of predominantly consumed food items by study area in Ethiopia, 2020.

Food items	Study areas, (*n*)	Mean	Min	Max	SD	*P*-value
Injera with legume-based stew	East Amhara, (n = 110)	17.78^a^	1.6	46.9	9.5	<0.001
	West Amhara, (*n* = 86)	3.34^b^	0.6	20.1	2.5	
Pasta/macaroni	East Amhara, (*n* = 77)	9.4^a^	1.0	33.58	6.8	0.007
	West Amhara, (*n* = 28)	8.75^a^	2.0	17.3	3.56	
Gruel/Porridge	East Amhara, (*n* = 19)	^‡^8.2 (2.1, 16.7)^a^	0.27	89.8		<0.001
	West Amhara, (*n* = 70)	1.6 (0.5, 3.5)^b^	<LOD	116.3		
Bread	East Amhara, (*n* = 43)	7.4^a^	1.7	20.81	4.05	0.144
	West Amhara, (*n* = 24)	1.4^b^	<LOD	3.2	0.84	
Cow’s milk (μg/L)	East Amhara, (*n* = 17)	0.34^a^	0.16	0.70	0.15	<0.001
	West Amhara, (*n* = 5)	0.21^b^	0.10	0.35	0.09	
Breast milk (μg/L)	East Amhara, (*n* = 51)	12.2^a^	<LOD	20.2	3.9	<0.001
	West Amhara, (*n* = 73)	3.4^b^	1.3	7.4	1.56	

Means with different superscripts are significantly different at 0.05. ^‡^Such values are median [Q_25%_, Q_75%_]. <LOD, Less than the limit of detection.

### Se intake

The mean Se intake of children from East Amhara was significantly higher than that from West Amhara on both days when samples were collected (Table 4). The total Se intake among children from East Amhara and West Amhara was 30.2 [IQ _25%,_14.2; IQ _75%_,54.1] and 7.4 [IQR _25%_,4.2; IQ _75%_, 10.6] μg day^–1^, respectively (Table 5); 31.5% of the study subjects from East Amhara and 92% from West Amhara had inadequate Se intakes. Daily Se intake variation was not significant between the two measurement days. There was no significant within-days variation in Se intake or meal frequency (3.5 vs. 3.6 meals per day). The mean Se intake from breast milk in East Amhara (6.8 μg/day) was significantly higher than that in West Amhara (1.9 μg/day), *p* < 0.001.

**TABLE 5:** Comparison of Se intake (μg/day) among infants between two measurement days from East Amhara and West Amhara.

	East Amhara (*n* = 74)	West Amhara (*n* = 75)
Day 1	25.4 (11.9, 46.1)^a^	7.1 (3.6, 10.3)^b^
Day 2	29.8 (12.5, 52.2)^a^	6.5 (3.2, 11.7)^b^

Numbers with different superscripts in the same row are significantly different at 0.01.

### Urine Se concentration

Urinary Se excretion comprised 53 and 39% of daily Se intake, respectively. Urine Se concentrations were significantly higher among study children from East Amhara compared with West Amhara (22.5 ± 11.5 μg/L vs. 3.0 ± 1.9 μg/L, *p* < 0.001). Overall, Se intake was positively correlated to urinary Se excretion (*r* = 0.56; *p* < 0.001); however, the correlation between dietary Se intake and urinary Se excretion among the study subjects from West Amhara was not significant (*r* = 0.16, *p* ≥ 0.05).

## Discussion

The present study assessed Se concentrations in complementary foods, breast milk, and the urine of children (aged 6–23 months) from two areas in Ethiopia. Consistent with previous reports of Se spatial variability in soils, crops, humans, and livestock in Ethiopia (11–14), most food items, breast milk, and urine samples analyzed from East Amhara had significantly higher Se concentrations compared with the samples from West Amhara. These results also agree with previous studies showing none or lower Se deficiency prevalence (0 to 41%) among populations in East Amhara but a higher prevalence (91.1%) in West Amhara (22).

There is large variation in the Se content of foods as affected by geographical location, environment, and soil factors. A review of published literature on the Se concentration in 148 food items reported that food items from the United States generally had the greatest Se content while foods from the United Kingdom and New Zealand had lower Se concentrations (23). That same study also reported the presence of Se variability in foods from the same country and regions. Similarly, in the present study, food items from East Amhara had high Se concentrations compared with food items from West Amhara. Also, human breast milk and cow milk samples showed a similar pattern of Se concentration. In addition, compared with the age reference dietary Se intake by Kipp et al. (21), children from West Amhara had a low median Se intake, 7.4 [IQR _25%_, 4.2; IQ _75%_, 10.6] μg day^–1^, and 92% of the participants had an inadequate Se intake, but those from East Amhara had a median Se intake of 30.2 [IQ _25%,_ 14.2; IQ _75%_, 54.1] μg day^–1^ with an inadequate Se intake prevalence of 31.5%. This is consistent with the findings of previous studies on cereal grain and soil (11, 12), and human (20, 22) and livestock (14) blood from the two areas. Significant variations in Se content across food groups are known, with animal-source foods such as fish, meat, and eggs having greater Se concentrations than cereals, and fruits and vegetables having the lowest Se concentrations (23, 24). The food items in the present study were dominated by cereal-based foods and consumption of food from animal sources was very limited.

The release of essential minerals such as Cu, Fe, and Zn into breast milk from the body is typically independent of maternal mineral status and is regulated by the mammary gland (25). However, in the present study, maternal Se status was positively correlated with breast milk Se concentration indicative of an absence of homeostatic regulation (26). In the present study, lower breastmilk Se was found among mothers from West Amhara, an area known for Se deficiency (11, 12). WHO recommends the exclusive breast feeding of infants until the age of 6 months and the timely introduction of complementary feeding afterward (27). However, in the present study, 26% of study subjects had an early (before 6 months) or late (several days or months after 6 months) introduction of complementary feeding. The early or late introduction of complementary feeding is associated with lower breast milk production by the mother, the reduced absorption of essential nutrients by children, nutritional inadequacy, and an increase in infection rates (28).

The results of the present study showed that children from West Amhara obtain very low concentrations of Se both from breastmilk and complementary foods, suggesting a need to design and implement interventions to address the deficiency. The biofortification of staple cereals has been reported to be cost effective in Ethiopia (29). Animal-source foods are known to contain good amount of Se but are only infrequently consumed by the children in the present study, hence interventions to improve access to and utilisation of animal-source foods in complementary food preparation is important.

Urine is a major excretory route for Se, hence urinary Se concentration is considered a viable biomarker for assessing Se status at a population level (19). There is a direct correlation between dietary Se intake and urinary excretion. A study on the relationship between dietary intake and urinary Se excretion among Japanese adults reported urinary Se excretion of 73 and 77% of dietary intake in men and women, respectively, as assessed by 24 h recall (30). In the present study, urinary Se excretion was positively correlated with 24 h dietary Se intake (*r* = 0.56; *p* < 0.001). In the present study, the predictive rate of urinary Se was lower than in other studies often conducted in high Se areas. This suggests that there may be homeostatic regulation to conserve Se in a state of deficiency (31).

This study was based on the weighed records of food samples prepared in the households on 2 days, a method considered the most precise to quantify food intake (32). Biochemical markers are useful in the validation of dietary assessment methods (32); therefore, the inclusion of urinary Se in the present study increases the reliability of the Se dietary intake estimate. Spot urine concentration is confounded by factors such as fluid intake, diet, and exercise (33); hence, in the present study, specific gravity adjustment was used to reduce the influence of these factors on our urinary Se estimate. The availability and accessibility of foods is strongly influenced by seasons (34). For example, the diversified diet availability to household members during autumn (post-harvest season) in Ethiopia is greater than other seasons (35). However, the present dietary assessment was limited to autumn and did not take into account seasonal variations or adjust for differences and therefore should be interpreted with caution.

In general, there was lower Se concentrations in food items, breast milk, and urine samples from West Amhara than East Amhara. Breast milk Se seems not to be subjected to homeostatic regulation, unlike other micronutrients such as Zn where their release into the breastmilk is favored. There was a positive correlation between dietary Se intake and urinary Se excretion among children from East Amhara but the relationship was not significant among participants from West Amhara, suggesting that the body has an ability to conserve Se when intake is inadequate. The children in the present study were from subsistent farming households that depend upon own/local food production; thus, the implementation of targeted interventions, such as agronomic biofortification of food crops widely consumed by the local population, could be important. In addition, because the analyzed foods had different Se concentrations, community nutrition education to select food items with better Se content (without compromising the intake of other nutrient) during food preparation is essential.

## Data availability statement

A de-identified, raw dataset used for this study will be shared upon reasonable request.

## Ethics statement

The studies were conducted in accordance with the local legislation and institutional requirements and approved by the National Research Ethics Review Committee at the Ministry of Science and Technology, Ethiopia (Reference 3.10/433/06) and Addis Ababa University Institutional Review Board (Reference CNSDO/450/10/2018). Written informed consent for participation in this study was provided by adult participants, and by legal guardians/next of kin for child participants.

## Author contributions

KH, EJ, MB, and DG: conceptualization. KH: data collection and supervision. KH, EB, LW, and KD: sample preparation and analysis. KH and DG: statistical analysis and original draft preparation. All authors contributed to writing - review and editing and have read and agreed to the published version of the manuscript.
